# The IL-20RB receptor and the IL-20 signaling pathway in regulating host defense in oral mucosal candidiasis

**DOI:** 10.3389/fcimb.2022.979701

**Published:** 2022-09-26

**Authors:** John E. Beute, Alex Y. Kim, Jamie J. Park, Allen Yang, Keshia Torres-Shafer, David W. Mullins, Paula Sundstrom

**Affiliations:** ^1^ Dartmouth College, Hanover, NH, United States; ^2^ Department of Microbiology and Immunology, Geisel School of Medicine at Dartmouth, Hanover, NH, United States

**Keywords:** oral candidiasis, IL-20 signaling pathway, keratinocytes, tissue homeostasis, IL-20RB

## Abstract

Pseudomembranous candidiasis (thrush), erythematous candidiasis, and fungal esophagitis are infections of the barrier mucosa of the upper gastrointestinal tract. The majority of these infections are caused by *Candida albicans*, an opportunistic fungal pathogen that frequently exists as a harmless commensal on mucosal surfaces lining the gastrointestinal tract. Oral infections are initiated in the superficial stratified squamous epithelium, in which keratinocytes are the most abundant host cells and are the initial points of contact with *C. albicans* present in saliva. Intrinsic features of oral keratinocytes are likely to play important roles in host defense and tissue homeostasis in oral candidiasis. One understudied pathway that may be important for modulating oral candidiasis is the IL-20 cytokine signaling pathway that employs keratinocyte IL-20RB receptors as ligands for IL-19, IL-20, and IL-24. We report that production of human oral keratinocyte *il24* mRNA and protein are stimulated during co-culture with *C. albicans*. To test the role of the IL-20 family signaling pathway in oral candidiasis, *Il20rb^-/-^* mice (lacking the IL-20RB receptor) were compared to wild-type mice in a murine model of oropharyngeal candidiasis. Fungal burdens and percent loss in body weight were determined. Despite comparable fungal burdens, the *Il20rb^-/-^* mice exhibited less weight loss over the course of their infection compared to the B6 mice, suggestive of reduced overall disease consequences in the mutant mice. Interference with IL-20 family cytokine signaling may be useful for augmenting the ability of the host to defend itself against pathogens.

## Introduction

Candida species are prominent components of the oral mycobiome of healthy adults (Fonzi and Irwin, 1993) and are also a common source of oral infections (Brown et al., 2012). Pseudomembranous candidiasis (thrush), erythematous candidiasis, and esophagitis are irritating infections of the barrier surfaces of human hosts. Oral candidiasis occurs with the onset of decreased immunity, which can be caused by a number of conditions such as HIV infection, immunosuppressive therapy, immature immunity in infants, and normal flora disruption by antibiotic treatment [Candida infections of the mouth, throat, and esophagus (2021); Bliss et al., 2012]. Although treatable, these conditions reduce the quality of life and are estimated to afflict one million people worldwide [How common are fungal diseases? (2022)] with annual treatment costs topping $335 million in the USA, alone (Benedict et al., 2019). A better understanding of host defenses and tissue repair processes is needed to develop new strategies for better management of these infections.

Oral candidiasis is most often caused by *Candida albicans*, a commensal pathogen that can invade the oral cavity, which consists of layers of keratinocytes in different stages of differentiation along with immune cells and resident bacteria bathed in saliva. Whereas IL-1-, neutrophil-, and IL-17-dependent mechanisms function in clearance (Conti et al., 2009; Huppler et al., 2014; Altmeier et al., 2016; Conti et al., 2016), they can also exert harmful effects on mucosal surfaces (McGeachy et al., 2019). How homeostasis is re-established, along with pathogen clearance, constitutes a critical gap in knowledge pertaining to acute oral candidiasis.

In particular, the role of oral keratinocytes of the stratified squamous epithelium—which form the primary host barrier defense in the oral mucosa and undergo pathologic alterations during candidiasis (Reichart et al., 2000)—is incompletely understood. Keratinocytes sense the presence of invading fungi and, like immune cells, secrete cytokines that recruit IL-17-producing cells and neutrophils that are critical for fungal clearance in murine and human oral mucosal infections (Trautwein-Weidner et al., 2015; Naglik et al., 2017). Superficial, differentiated keratinocytes express IL-17 receptors to activate antifungal defenses in the presence of *C. albicans* (Conti et al., 2009; Naglik et al., 2014). Therefore, studies on intrinsic keratinocyte signaling pathways that facilitate communications with immune cells are likely to yield important information about mechanisms of pathogen clearance and return to tissue homeostasis.

A newly recognized keratinocyte intrinsic signaling pathway is the IL-20 cytokine family signaling pathway (Rutz et al., 2014). Very little is known about the role of this pathway in host responses to pathogens in general. The IL-20 cytokine signaling pathway has been shown to be important in the communication between epidermal keratinocytes and canonical immune cells (Kunz et al., 2006; Sabat, 2010; Madouri et al., 2018). IL-20RB dimerizes with either IL-20RA or IL-22RA. These IL-20RB (also termed IL-20R2)-containing heterodimers, which are found on keratinocytes and neutrophils but not on T cells, B cells, or monocytes (Kunz et al., 2006; Ippagunta et al., 2016; Gough et al., 2017; McGeachy et al., 2019), serve as receptors for IL-19, IL-20, and IL-24. Their potential importance in the biology of the epidermal stratified squamous epithelium and barrier function is consistent with: (1) *in vitro* studies using reconstituted human epithelium (Sa et al., 2007); (2) their high expression in human skin and esophagus [National Library of Medicine (US), 1988]; and (3) the human genome analyses that link them to functions in psoriasis, inflammatory bowel disease, suppression of melanoma (IL-24 only) (GeneCards®: The Human Gene Database, 2022) and regulation of epidermal inflammation (Jin et al., 2014). Cytokines that signal through IL-20RB have been shown to promote cutaneous and pulmonary infections caused by *S. aureus* and *S. pneumococcus* (Myles et al., 2013; Madouri et al., 2018). It has been suggested that the IL-10-like roles of the IL-20 family cytokines (IL-19, IL-20 and IL-24), while enhancing repair processes at epithelial surfaces, may adversely impact pathogen clearance (Sabat, 2010; Madouri et al., 2018; Kirchner et al., 2019). Recently, IL-20RB receptors have also been found to be upregulated on human neutrophils, reducing cytotoxicity in the presence of *S. aureus* and lessening the damaging effects of inflammation (Gough et al., 2017). To our knowledge, there are no reports regarding the role of IL-19, IL-20, or IL-24 cytokines in fungal infections.

Our focus on oral keratinocyte signaling is also important because most research on the activity of the IL-20 signaling pathway in keratinocytes has been performed with *epidermal* keratinocytes. Both oral and epidermal keratinocytes have similar barrier functions, differentiation programs, structural features, and interactions with supporting tissue layers; however, stratified squamous epithelia formed by oral keratinocytes exhibit greater wound healing and proliferative properties compared to epidermal keratinocytes (Turabelidze et al., 2014), suggesting that there may be oral keratinocyte-specific properties of IL-20 family signaling that differ from those of epidermal keratinocytes.

The surprising finding that IL-20RB-deficient mice exhibited increased resistance to staphylococcal cutaneous infections, coupled with the downregulation of protective IL-17 responses (Myles et al., 2013), prompted us to ask whether this receptor might be playing a similar role in oral candidiasis. In the case of cutaneous staphylococcal infections in wild-type mice, IL-20RB signaling in epidermal keratinocytes (particularly *via* the IL-24 ligand) effectively dampened protective IL-17 responses leading to larger epidermal lesions compared to IL-20RB-deficient mice, which exhibited higher levels of IL-17 and Th17 cells. *C. albicans* and *S. aureus* are both opportunistic pathogens that reside within stratified squamous epithelia and, thus, may elicit similar interactions between epithelia and immune processes. Studying the role of the IL-20RB receptor will increase our knowledge of the role of the IL-20 signaling pathway in mucosal disease and our understanding of the *C. albicans* host-pathogen interplay in the oral cavity.

## Materials and methods

### Mice and ethical review

Wild-type C57BL/6J were purchased from Jackson Laboratories (Bar Harbor, Maine). Mating pairs of *il20rb^-/-^* mice on the C57BL/6 background (a generous gift from Sandip K. Datta, M.D. at the NIH) were bred at the Center for Comparative Medicine and Research at Dartmouth College. All protocols were approved by the Institutional Care and Use Committee at Dartmouth College.

### Mouse model of oropharyngeal candidiasis (OPC)

The OPC model was performed as described (Solis and Filler, 2012; Conti et al., 2014) with minor modifications. Briefly, mice were age- and sex-matched in groups of 6–8 animals and were infected without immunosuppression unless otherwise indicated. For oral inoculation, mice were first anesthetized using a cocktail of ketamine:xylazine (100 mg/kg and 5–10 mg/kg, respectively) and then orally inoculated with *C. albicans* strain CAF2-1 (Fonzi and Irwin, 1993) or its oral-adapted lineages (see below). Inoculation was accomplished by inserting a 0.0025-g cotton ball saturated with 2 × 10^7^ cells sub-lingually for 75 min, as previously described (Conti et al., 2014). Mice were weighed daily for four days. To assess fungal burdens, tongues from euthanized mice were removed, weighed, and homogenized in sterile PBS using a Pro200 Bio-Gen Homogenizer (PRO Scientific, Oxford, Connecticut) prior to dilution and plating (Solis and Filler, 2012) onto Sabouraud’s dextrose agar containing chloramphenicol. Colony-forming units/g tissue were determined.

### Oral-adapted lineages

To elicit OA lineages, CAF2-1 was serially passed through the OPC model three times. This was accomplished by first culturing tongue homogenates harvested two days after inoculation on YPD media containing chloramphenicol. The lawn of cultured organisms was pooled and used to inoculate a second mouse, from which organisms were harvested and used to inoculate a third mouse. Three individual colonies were isolated from tongue homogenates of the third mouse and lineages were named OA1, OA2, and OA3, respectively, and stored at -80°C.

### *C. albicans* strains, culture, and handling

*C. albicans* strains were used in this study. The strains used in this study include SC5314 and its derivatives, CAF2-1 and UnoPP-1; they have all been shown to elicit loss in murine body weight during oral candidiasis (Conti et al., 2009; Schönherr et al., 2017) and **Supplementary Figure S1**. All strains were stored at -80°C in media supplemented with glycerol. Prior to use in the OPC assay, strains were grown on YPD agar plates for 48 hours followed by inoculation into liquid YPD at 30°C with shaking at 250 rpm for 14–18 hours.

### Statistical methods

To determine the significance of differences in percent weight loss by day between *il20rb^-/-^* and wild-type B6 mice a longitudinal analysis using a linear mixed model with random-subject effect was used. The outcome was percent of weight loss from Day 0. Covariates included in the model were group, Day, and their interaction, where Day was treated as a categorical variable. Least square means (LSM) and its 95% confidence interval by group and Day from the model was provided. Finally, the outcome was compared between the two groups on each Day and P-values were calculated for significance. All statistical analyses were conducted in SAS 9.4 (Cary, NC, SAS Institute Inc.), with a two-sided significance level of 5%. The analysis was performed by Zhongze (George) Li in the Biomedical Data Science Department at Dartmouth College.

The statistical test for the differences in IL-24 and IL-8 levels in the presence and absence of UV-treated *C. albicans* germ tubes was the two-sample *t*-test assuming equal variances, using the Microsoft Excel 16.16.1 Data Analysis ToolPak.

### Microarray and qRT-PCR

RNA was prepared from co-cultures of *C. albicans* UnoPP-1, which was derived from strain SC5314 (Postlethwait and Sundstrom, 1995) and OKF6/TERT-2 keratinocytes described in a previous study (Rollenhagen et al., 2009). Two RNA samples were prepared for each 0-, 3-, 6-, and 12-hour time point. Lysates of co-cultures were treated as described in the Qiagen RNeasy Mini handbook for animal cells, including DNase digestion. The concentration and the purification grade of the eluted RNA was determined with a Nanodrop photometer. The 260/280 and 260/230 ratios were between 1.9 and 2.5, respectively, indicating a high purity of the RNA. After re-checking its purity on an Agilent Bioanalyzer, the RNA was hybridized to Illumina HumanHT-12 microarrays at the Genomics and Molecular Biology Shared Resources Facility at Dartmouth College. Analyses of the resulting Illumina GenomeStudio text files with non-normalized and non-background subtracted values were performed using Ingenuity iReport.

For qRT-PCR, human primary oral keratinocytes (Sciencell Research Laboratories, Carlsbad, DA) were propagated in Oral Keratinocyte Medium (OKM) at 37°C in an atmosphere containing 5% CO_2_. Cells were trypsinized, pelleted, and dispensed into 12-well tissue culture plates at a concentration of 2 x 10^5^ cells/well in 1 mL of OKM media and incubated overnight prior to the addition of cells from an overnight culture of *C. albicans* strain SC5314 at an MOI of 0.1 or 1.0. After 2 hours of co-culture, rhTNF-2 from R&D systems with BSA carrier (10 ng/well) was added and incubation was continued for a total of 12 hours. qRT-PCR was performed on RNA prepared as described above in duplicate using TaqMan and normalization to GAPDH.

### Detection of IL-24 and IL-8

Co-cultures were prepared as described above except that UV-treated SC5314 germ tubes were used to inactivate fungi with minimal alteration to fungal surface properties that may be important for eliciting keratinocyte IL-24 expression. Inactivation of fungi permitted the use of longer incubation periods allowing keratinocytes to maximize production of IL-24 without being damaged by fungal overgrowth. Germ tubes were prepared by inoculating pre-warmed M199 with an overnight culture of SC5314 grown in yeast nitrogen broth (YNB) medium (Rollenhagen et al., 2009) incubated at 30°C and shaken at 250 rpm. After incubation for 3 hours in M199, the germ tubes were washed in ice-cold PBS and UV treated (Wheeler and Fink, 2006). UV-treated germ tubes (50 uL/well in PBS) were added to keratinocytes in 12-well plates at an MOI of 10:1 and incubated for four hours prior to adding cytokines to cultures as follows: rhTNF-2 from R&D systems with BSA carrier (10 ng/well) and an rhIL-17A/F heterodimer carrier free from BioLegend (100 ng/well). Co-cultures were incubated for an additional 48 hours. Supernatants were harvested and assayed for human IL-24 in duplicate using the DuoSet ELISA Development System from R&D Systems and for IL-8 using BioLegend’s ELISAMAX for Human IL-8 according to the manufacturer’s directions.

## Results

### Expression of IL-24 in human oral keratinocytes in response to *C. albicans*

To determine if human oral keratinocytes produce IL-24 in response to *C. albicans*, we performed gene expression analysis and qRT-PCR using mRNA collected from co-cultures of either the human oral keratinocyte cell line OKF6/TERT-2 cells (Rollenhagen et al., 2009) or primary human oral keratinocytes (**Supplementary Table 1**). We found that *il24* mRNA was induced within 6 hours of co-culture of *C. albicans* with OKF6/TERT-2 or primary human oral keratinocytes (**Supplementary Table 1**).

**Figure 1:**
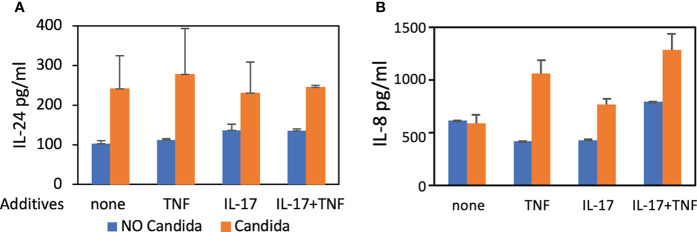
ELISA. Detection of IL-24 and IL-8 protein levels following co-culture of UV-treated *C. albicans* SC5314 germ tubes and primary human oral keratinocytes (MOI 10:1) for 48 hours in the presence of TNF (10 ng/mL) and/or IL-17 (100 ng/mL). **(A)** The amount of IL-24 secreted in the presence of germ tubes (M = 248.5 pg/mL) was significantly greater than controls (M = 121.9 pg/mL) *P* < 0.05. **(B)** The amount of IL-8 was increased in the presence of germ tubes and TNF (M=1062.6 vs. 423.9 pg/mL) or IL-17 (M=772.7 vs. 435.53 pg/mL) or both TNF and IL-17 (M=1289 vs. 796.5 pg/mL) *P* < 0.05 but not in the presence of germ tubes alone (M=596.2 vs. 622.4 pg/mL) *P* = 0.8.

We also found IL-24 to be 1.7-fold increased over controls 48 hours after stimulation with UV-treated *C. albicans* germ tubes (**Figure 1A**) using ELISA. *C. albicans* induces human oral keratinocytes to synthesize and secrete IL-24 even in the absence of TNF and IL-17, which augment the synthesis of pro-inflammatory components such as IL-8 (Zenobia and Hajishengallis, 2015). To show that these co-cultures are secreting cytokines consistent with previously published models, we also demonstrated that IL-8 production was increased in the presence of *C. albicans* as expected (**Figure 1B**), although unlike results from other studies (Dongari-Bagtzoglou and Kashleva, 2003), stimulatory cytokines were required.

### Mice deficient in the IL-20RB receptor exhibit equivalent fungal burdens but reduced weight loss during acute oropharyngeal candidiasis (OPC)

**Figure 2:**
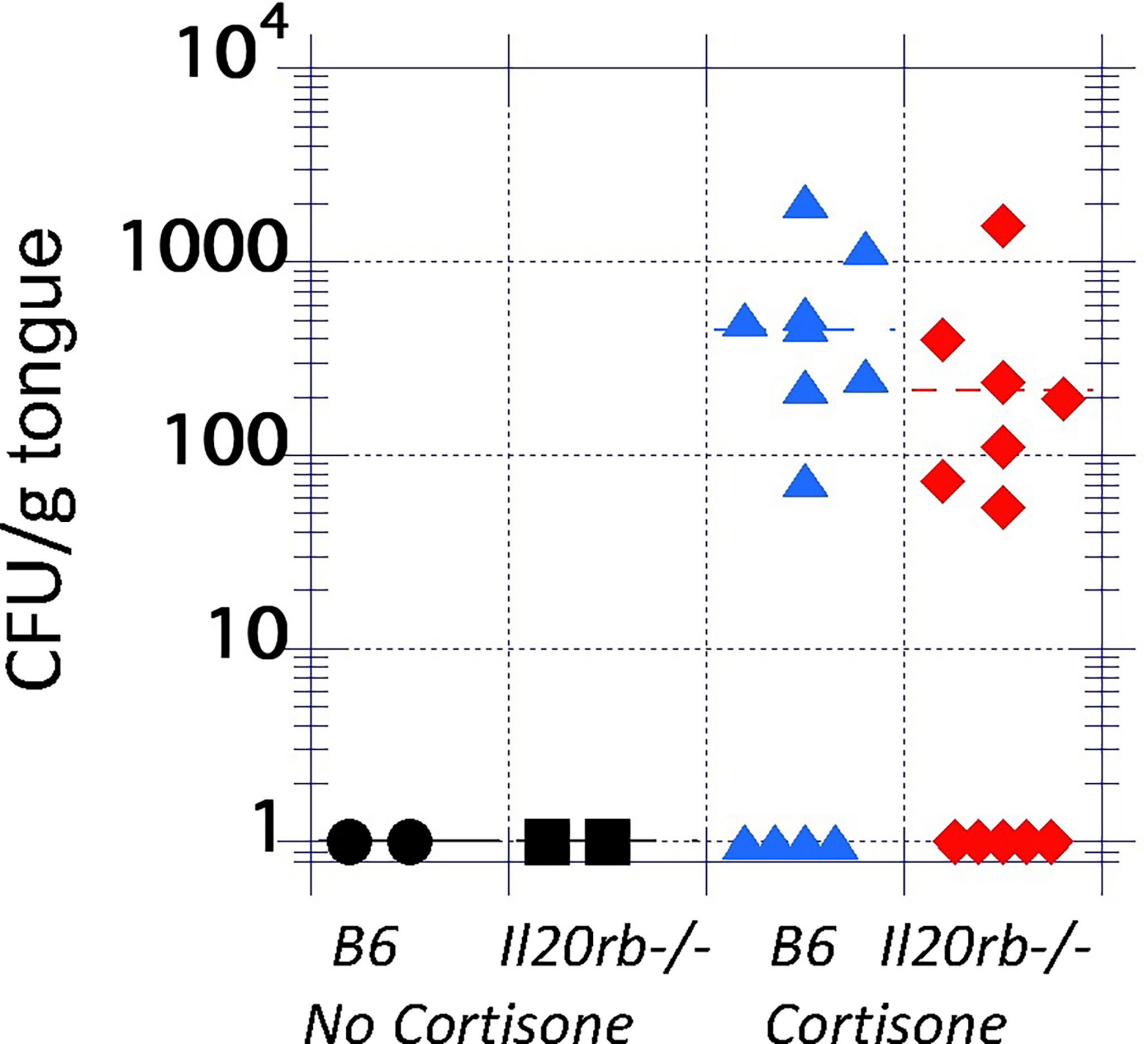
Fungal burdens in the tongues of untreated and immunosuppressed mice on Day 4 after inoculation with *C. albicans* strain CAF2-1. Infections were cleared in the absence of cortisone in both groups (black markers). In the presence of low-dose cortisone (17.5 mg/kg) to extend the infection, as previously described (Simpson-Abelson et al., 2015), the differences in fungal burdens between *il20rb^-/-^* (red diamonds) and wild-type B6 (blue triangles) mice were not statistically significant. Each marker represents one mouse. See the Materials and Methods section for a description of the statistical analysis.

To test the role of the IL-20 family signaling pathway in oral candidiasis, mice deficient in the IL-20RB receptor were obtained from the NIH. These mice were generated as described previously (Zheng et al., 2008) and were used in prior studies showing increased resistance to staphylococcal cutaneous infections (Myles et al., 2013). Infection was initiated and showed a mean of 8 x 10^4^ CFU/g tongue on Day 1 with complete clearance by Day 4 (**Supplementary Figure S2**), similar to that described for B6 mice (Conti et al., 2009). IL-20RB was not required for clearance (**Figure 2**, black circles, and squares). To determine if IL-20RB played a role in promoting infection, as was seen in staphylococcal cutaneous infections, it was necessary to generate conditions in which fungi persisted beyond 4 or 5 days. To do this, the mice were immunosuppressed to achieve a low fungal burden using a low dose of cortisone (see discussion for rationale), as previously described (Simpson-Abelson et al., 2015) (**Figure 2**). Fungal burdens in the tongue were equivalent in both groups of mice.

**Figure 3:**
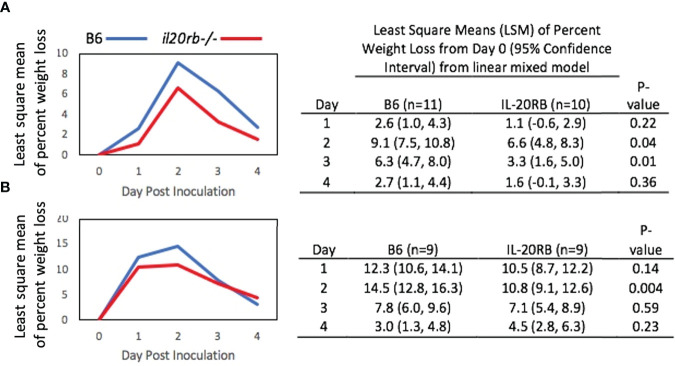
Percent weight loss relative to Day 0 in *il20rb-/-* and WT B6 mice after inoculation with *C. albicans* CAF2-1. Mice deficient in the IL-20RB receptor (red) exhibit reduced weight loss during oral candidiasis compared to WT B6 mice (blue), matched for age and gender. Low-dose cortisone (15 mg/kg) was used in the experiment shown in **(A)** but not in **(B)**. The mean percent losses in body weight in *il20rb-/-* mice were lower than those of WT B6 mice on Days 2 and 3 in part A and on Day 2 in part **(B)** See the Materials and Methods section for a description of the statistical analysis. The raw data are provided in **Supplementary Table S2**.

Despite having similar fungal burdens, the *il20rb^-/-^* mice exhibited less weight loss over the course of infection compared to the B6 mice (**Figure 3A**). A second experiment performed in the absence of cortisone showed similar results (**Figure 3B**). A least-square-means longitudinal analysis showed that the percent weight loss from Day 0 was significantly different between the two groups of mice on Days 2 and 3 (*P*-values = 0.04 and 0.01) in **Figure 3A**, and on Day 2 (*P*-value = 0.004) in **Figure 3B**.

**Figure 4:**
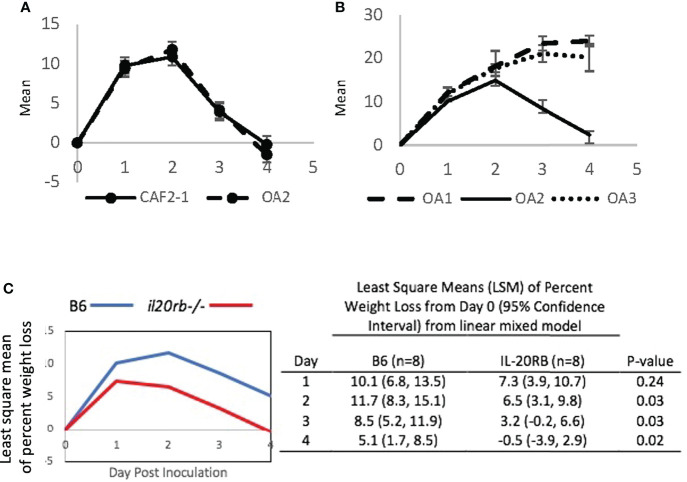
Mean percent loss in body weight caused by oral-adapted (OA) CAF2-1 lineages in wild-type B6 mice during acute oral candidiasis, relative to Day 0 post-inoculation. **(A)** The weight loss profile of OA2 was identical to that of the parental strain CAF2-1. **(B)** OA1, OA2, and OA3. Differences between OA1 and OA2, and OA3 and OA2 were statistically significant on Days 3 and 4 post inoculation (*P* < 0.01). Each experiment was performed once with an *N* = 4. **(C)** Percent weight loss relative to Day 0 in *il20rb^-/-^* and wild-type B6 mice after inoculation with *C. albicans* strain OA1. Mice deficient in the IL-20RB receptor (red) exhibited reduced weight loss on Days 2, 3, and 4 during oral candidiasis compared to wild-type B6 mice (blue), matched for age and gender. See the Materials and Methods section for a description of the statistical analysis. The raw data are provided in **Supplementary Table S2**.

Serial passaging experiments of single-colony isolates in the CAF-2 genetic background produced three oral adapted (OA) lineages that were tested in the OPC model (**Figures 4A, B**). Infection by OA1 and OA3 increased weight loss compared to OA2, which caused weight loss kinetics identical to CAF2-1. When challenged with OA1, which causes increased weight loss compared to the parental strain CAF2-1, the *il20rb^-/-^* group not only recovered its initial mean weight but showed weight gain on Day 4, indicative of recovery from infection (**Figure 4C**). In contrast, the wild-type B6 group, which normally clears infections by Day 4 or 5 using strain CAF2-1, sustained a 5% mean weight loss on Day 4 when inoculated with OA1. Furthermore, the magnitude of the difference between the two groups of mice on all days was greater when the OA1 lineage, rather than the parental strain (**Figure 3**), was used. The *il20rb^-/-^* mice were better able to maintain weight in the presence of OA1, which elicited higher losses in body weight compared to B6 mice. The results support the hypothesis that inhibition of protective immunity by IL-20 signaling in B6 mice is detrimental to recovery from candidiasis, especially in the presence of strains with elevated virulence.

## Discussion

Our previous research on the interaction of *C. albicans* and human oral keratinocytes revealed dynamic effects on keratinocyte cytoskeletal reorganization, motility, and differentiation (Rollenhagen et al., 2009) that raised questions about the roles of keratinocytes in host defense. The exciting discovery that IL-22 binds receptors on oral basal keratinocytes to increase IL-17 receptors in superficial keratinocytes is an enlightening example of how the IL-20 signaling pathway functions across complex stratified epithelium layers to modulate host defense. Furthermore, the IL-22-mediated upregulation of IL-17 receptors is a host response that targets the keratinocytes that are being induced to differentiate by invading fungi (Rollenhagen et al., 2009).

A key unanswered question in the pathological response to oral candidiasis concerns the regulation of IL-17-mediated protective immunity—both in acute candidiasis (addressed in this study) and also in the attenuation of the inflammatory response in commensalism (Kirchner et al., 2019). Not much is known about the function of the IL-19, IL-20, and IL-24 cytokines and their receptors, which are intrinsic to keratinocytes and are mostly associated with autoimmunity in psoriasis (Sa et al., 2007). The little that is known regarding responses to microbial pathogens (Myles et al., 2013; Madouri et al., 2018) suggests a role in keratinocyte-mediated regulation of protective IL-17-mediated immunity, which prompted us to investigate this possibility in oral candidiasis. We found that primary human oral epithelial cells, as well as immortalized OKF6/TERT2 cells, express *il24* mRNA and secrete IL-24 following exposure to *C. albicans*. *il24* is also expressed in reconstituted oral human epithelium following incubation with *C. albicans* (Moyes et al., 2014). In *IL-17RA^KO^* mice in the OPC model, *il24* mRNA expression in the tongue tissue was found to be decreased compared to wild-type mice, suggestive of an interaction between the IL-20 and IL-17 signaling pathways (Conti et al., 2009). These results are consistent with a possible role for IL-20 signaling during oral keratinocytes in oral candidiasis in mice and humans.

To gain further evidence for the role of the IL-20 signaling pathway in the protection or exacerbation of acute oral candidiasis, we assessed tongue fungal burdens in IL20RB-deficient mice in OPC. Using a low dose of cortisone (Simpson-Abelson et al., 2015), we established a low level of infection that extended to 4 to 5 days of the model so that we would be able to detect reduced burdens in the mutant mice. Unlike the reduction in bacterial burdens in staphylococcal cutaneous infections in the *il20rb^-/^* ^-^ compared to wild-type mice, the fungal burdens between the wild-type and mutant mice were equivalent. This finding does not necessarily rule out differences in the severity of oral candidiasis between groups, though, as fungal burdens have not been correlated with transient losses in body weight (Schönherr et al., 2017). On the other hand, the IL-20RB-deficient mice exhibited a modest yet highly reproducible reduction in the amount of transient loss in body weight, which is a reflection of disease severity. The exact cause of loss in body weight during OPC is complex and may be related to the damage associated with a rapid inflammatory response following inoculation, along with difficulty in eating, esophageal blockage, and/or alteration of the microbiome. It is also important to point out that the mutant mice lack the IL-20RB receptor in all tissues, not just in oral keratinocytes. Therefore, it is possible that the reduced weight loss observed in the mutant mice may be an indirect consequence of undescribed roles of IL-20 in cells other than oral keratinocytes. In any case, the decreased loss in body weight in the mutant mice reflects reduced disease severity. The study by Schönherr et al. (Schönherr et al., 2017) also showed that loss in body weight is correlated with PMN levels in the tissue. Our histological studies, however, failed to show differences in the PMN levels between the two groups of mice.

Our results in combination with previous work on IL-22 suggest possible roles for both activation and regulation of immunity in OPC. Unlike IL-20RB family cytokines (IL-19, IL-20, and IL-24), IL-22 binds to IL-20R1 or IL-22R1 dimerized with IL-10R2 receptors and contributes to protective host defenses for several pathogens including *C. albicans* (Eyerich et al., 2011; Bichele et al., 2018). Recent research has shown that IL-22 promotes epithelial defenses in general by activating appropriate niche-specific effector mechanisms (Tsai et al., 2017; Wang et al., 2017). The exciting discovery that IL-22 binds receptors on oral basal keratinocytes, which leads to an increase in IL-17 receptors on superficial keratinocytes (Aggor et al., 2020), is an example of how the IL-20 signaling pathway functions to modulate host defense in the complex stratified epithelium composed of keratinocyte layers in various stages of differentiation. In the gut, mucosal associated commensal fungi activate IL-22 production, which functions in protecting the gut mucosa from damage (Leonardi et al., 2022). Furthermore, the IL-22-mediated upregulation of IL-17 receptors is a host response that perfectly arms the mature keratinocytes that are on the front lines battling invading fungi (Rollenhagen et al., 2009).

These findings point to immune responses in oral candidiasis being simultaneously protective and damaging and underscore the need for tightly regulated control within the epithelium in oral candidiasis. Along these lines, the more pronounced reduction in body weight loss by IL-20RB-deficient (compared to wild-type) mice in the presence of the OA1 lineage, which induces more weight loss than the parental strain, is consistent with a role for the IL-20RB signaling pathway in preserving health when challenged by strains that are highly damaging to the host.

In summary, this study is consistent with the IL-20RB signaling pathway playing an important role in modulating the damaging effects of protective immunity to benefit the keratinocyte-rich environment of the oral epithelium. In addition, the literature reflects an emerging recognition of interactions between IL-17 and IL-20 signaling to protect epithelial surfaces from pathogens while repairing wounds and maintaining homeostasis (Tohyama et al., 2009; Kolumam et al., 2017; Xu et al., 2021). This study also prompts questions about the possible influence of IL-22 on IL-20RB receptor expression and its ligands that could potentially couple clearance to homeostasis of the oral-stratified squamous epithelium. More research is needed to localize and determine the dynamics of the expression IL-20 cytokines in the oral epithelium and how they impact inflammation, loss in body weight, and tissue repair in oral candidiasis.

## Data availability statement

The raw data supporting the conclusions of this article will be made available by the authors, without undue reservation.

## Ethics statement

The animal study was reviewed and approved by Institutional Animal Care and Use Committee - Dartmouth.

## Author contributions

JB, AK, JP, and AY conducted the OPC experiments and interpreted the data. KT-S designed and carried out the qRT-PCR experiments and interpreted the data. DM supervised the co-culture and qRT-PCR experiments and interpreted the data. PS devised research goals, designed, managed, and interpreted all experiments, wrote the manuscript, supervised and conducted OPC experiments, and acquired funding. All authors reviewed the manuscript. All authors contributed to the article and approved the submitted version.

## Funding

This research was supported by the Geisel School of Medicine at Dartmouth and a Pilot Award to PS from a COBRE award that funded the Center of Biomedical Research Excellence in Molecular, Cellular and Translational Immunology (Phase III), P20RR16437, P30GM103415, to William R Green (PI).

## Acknowledgments

We thank members of the CCMR at Dartmouth for excellent assistance and advice on the animal experiments including rodent handling, quarantining, and breeding the *il20rb^-/-^* mice, anesthesia, and presentation of results. We appreciate Heather Conti for consultation regarding the implementation of the OPC model, Christiane Rollenhagen for preparing the mRNA for the microarray experiments. We also appreciate valuable advice from the immunologists in the Department of Microbiology and Immunology at the Geisel School of Medicine at Dartmouth.

## Conflict of interest

The authors declare that the research was conducted in the absence of any commercial or financial relationships that could be construed as a potential conflict of interest.

## Publisher’s note

All claims expressed in this article are solely those of the authors and do not necessarily represent those of their affiliated organizations, or those of the publisher, the editors and the reviewers. Any product that may be evaluated in this article, or claim that may be made by its manufacturer, is not guaranteed or endorsed by the publisher.

## Supplementary material

Supplementary Figure 1 Percent weight loss relative to Day 0 in wild-type B6 mice after inoculation with *C. albicans* CAF2-1 and Unopp-1. Strains CAF2-1 and Unopp-1 caused similar losses in body weight in the OPC model. *N* = 2 males for mice that received Unopp-1, *N* = 3 females for mice that received CAF2-1. Differences in weight loss on Days 1, 2, 3, and 4 were not statistically significant.

Supplementary Figure 2 Fungal burden in tongue tissue of *il20rb-/-* mice on Days 1, 2, 3, and 4 following oral inoculation with CAF2-1. Each dot represents a single mouse.
