# Supplementary figures and images for: The IL-20RB receptor and the IL-20 signaling pathway in regulating host defense in oral mucosal candidiasis
Source: Front Cell Infect Microbiol. 2022 Sep 26;12:979701. doi: 10.3389/fcimb.2022.979701 (PMC9548646; doi:10.3389/fcimb.2022.979701)

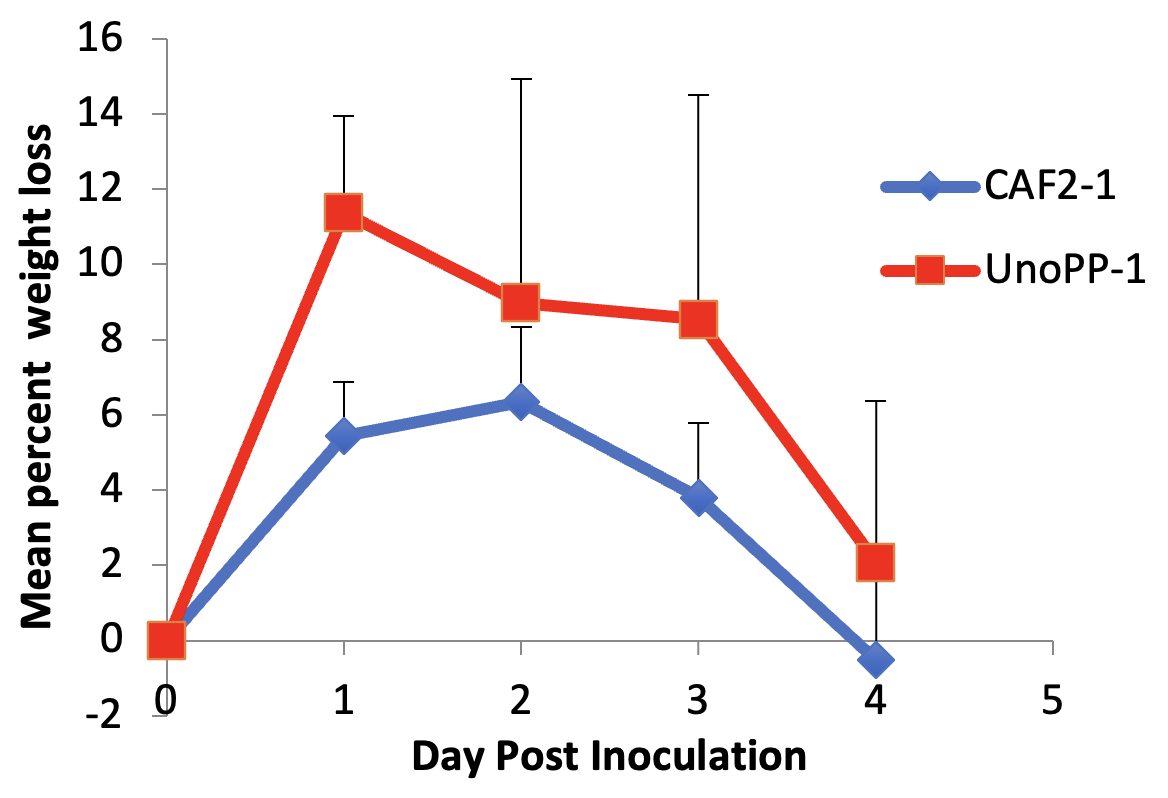

Supplement: Supplementary Figure 1 — Percent weight loss relative to Day 0 in wild-type B6 mice after inoculation with C. albicans CAF2-1 and Unopp-1. Strains CAF2-1 and Unopp-1 caused similar losses in body weight in the OPC model. N = 2 males for mice that received Unopp-1, N = 3 females for mice that received CAF2-1. Differences in weight loss on Days 1, 2, 3, and 4 were not statistically significant. [file Image_1.tiff]

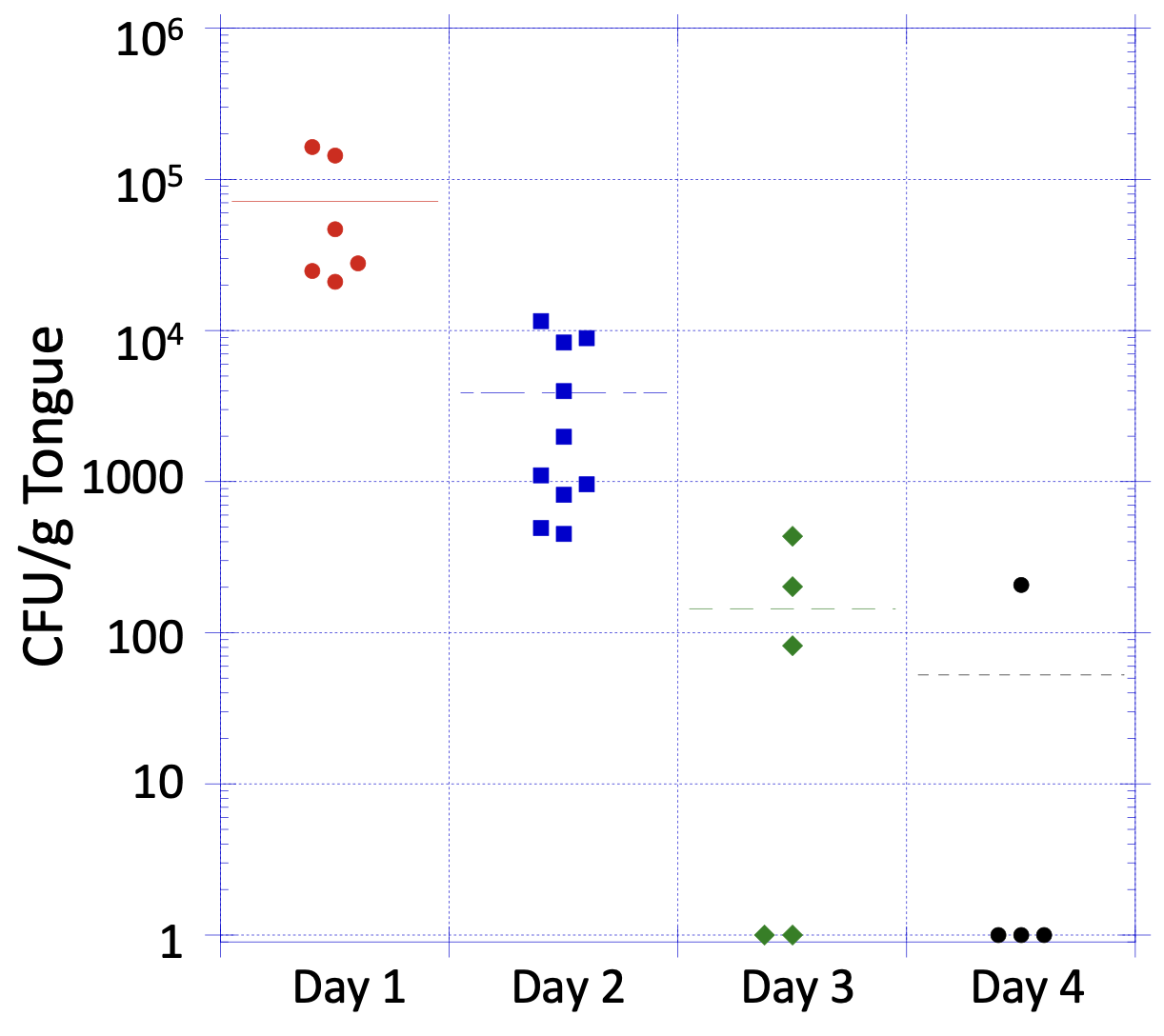

Supplement: Supplementary Figure 2 — Fungal burden in tongue tissue of il20rb-/- mice on Days 1, 2, 3, and 4 following oral inoculation with CAF2-1. Each dot represents a single mouse. [file Image_2.tiff]
