# Supplementary material for: The IL-20RB receptor and the IL-20 signaling pathway in regulating host defense in oral mucosal candidiasis
Source: Front Cell Infect Microbiol. 2022 Sep 26;12:979701. doi: 10.3389/fcimb.2022.979701 (PMC9548646; doi:10.3389/fcimb.2022.979701)
Supplement: Supplementary file 3 [file Table_1.docx]

Supplementary Table 1

Table 1. Induction of IL-24 gene expression in OKF6/TERT-2 (microarray) and primary human oral keratinocytes (qRT-PCR) by live *C. albicans*

| **Experiment** | **Hours of**  **co-culture** | **MOI** | **Fold**  **Change** | **Source** |
| --- | --- | --- | --- | --- |
| Microarray | 6 | 0.1 | 3 | This work |
| Microarray | 12 | 0.1 | 3.4 | This work |
| Microarray | 24 | unk | 4.3 | (Moyes et al.) |
| qRT-PCR | 12 | 0.1 | 14 | This work |
| qRT-PCR | 12 | 1.0 | 8 | This work |
